# Supplementary material for: Powerful extragalactic jets dissipate their kinetic energy far from the central black hole
Source: Nat Commun. 2020 Oct 30;11:5475. doi: 10.1038/s41467-020-19296-6 (PMC7588489; doi:10.1038/s41467-020-19296-6)
Supplement: Supplementary file 1 — Supplementary Information [file 41467_2020_19296_MOESM1_ESM.pdf]

Powerful extragalactic jets dissipate their kinetic energy far from the central black hole

Supplementary Information

Adam Leah W. Harvey*[1], Markos Georganopoulos[1,2], and Eileen T. Meyer[1]

[1]University of Maryland, Baltimore County, Department of Physics, 1000 Hilltop Cir Baltimore, Maryland, USA 21230
[2]NASA Goddard Space Flight Center
aharvey1@umbc.edu, georgano@umbc.edu, meyer@umbc.edu

1 Supplementary Information

1.1 Derivation of the Seed Factor

We assume here that gamma-ray emission is due to external Compton scattering. We begin with the peak energies (in units of electron rest mass energy) of the synchrotron and inverse Compton SED components, as seen in the observer's frame. These are, respectively,

$$\epsilon_s = \frac{B}{B_{cr}}\gamma^2\delta/(1+z) \tag{1}$$

$$\epsilon_c = \frac{4}{3}\epsilon_0\gamma^2\delta^2/(1+z) \tag{2}$$

where ϵ_0 is the characteristic energy of the external seed photons in units of the electron rest mass energy, B is the magnetic field in the jet, δ is the Doppler factor, γ is the Lorentz factor of the electrons responsible for the SED peak energy emission of both the synchrotron and inverse Compton SED components, and B_{cr} is the critical magnetic field ($B_{cr} = m_e^2c^3/e\hbar$).

Supplementary Equation (2) is valid if the electron scattering takes place in the Thomson regime, which holds if,

$$\nu_c \lesssim 3.5 \times 10^{23}/(1+z) \text{ Hz}. \tag{3}$$

Here, as in the rest of the paper we have assumed that the highest energy possible for external seed photons in the broad-line region and molecular torus are UV emission-line photons which have $\epsilon_0 \approx 3 \times 10^{-5}$ [1]. For powerful blazars, generally, $\langle\nu_c\rangle \approx 10^{22}$ Hz. Thus the peak of the inverse Compton component is generally produced in the Thomson regime for powerful blazars.

Dividing Supplementary Equation (2) by Supplementary Equation (1) and solving for B/δ,

$$\frac{B}{\delta} = \frac{4\epsilon_0\epsilon_s B_{cr}}{3\epsilon_c}. \tag{4}$$

We now consider the Compton dominance. This is the ratio of the observed peak inverse Compton luminosity, given by

$$L_c = \frac{16}{9}\sigma_T c\beta_p\gamma_p^2 n(\gamma_p)U_0\delta^6 \tag{5}$$

and the observed synchrotron peak luminosity, given by

$$L_s = \frac{4}{3}\sigma_T c\beta_p\gamma_p^2 n(\gamma_p)U_B\ \delta^4 \tag{6}$$

Here σ_T is the Thomson cross section, c is the speed of light, γ_p is the Lorentz factor of the electrons responsible for the peaks of the synchrotron and inverse Compton components, $n(\gamma_p)$ is the electron density distribution at γ_p, β_p is the speed of these electrons in units of c, Γ is the bulk Lorentz factor, U_0 is the external photon field energy density in the galaxy frame, and $U_B = B^2/8\pi$ is the magnetic field energy density. Their ratio is given by

$$k = \frac{L_c}{L_s} = \frac{32\pi\delta^2U_0}{3B^2}, \tag{7}$$

where U_0 is the energy density of the external photon field.

Solving for B/δ, equating to Supplementary Equation (4), and doing some simple algebra, we find

$$\frac{U_0^{1/2}}{\epsilon_0} = 3.22 \times 10^4 \, \frac{k_1^{1/2}\nu_{s,13}}{\nu_{c,22}} \text{ Gauss}, \tag{8}$$

where k_1 is the Compton dominance in units of 10, $\nu_{s,13}$ is the synchrotron peak in units of 10^{13} Hz, and $\nu_{c,22}$ is the EC peak in units of 10^{22} Hz.

This gives us the seed factor,

$$\text{SF} = \text{Log10}\left(\frac{U_0^{1/2}}{\epsilon_0}\right) = \text{Log10}\left(3.22 \times 10^4 \, \frac{k_1^{1/2}\nu_{s,13}}{\nu_{c,22}} \text{ Gauss}\right). \tag{9}$$

We note here that similar equations have been presented in [2] without discussing how these equations can be used to infer the precise location of gamma-ray emission in FSRQs.

1.2 Estimating the Seed Factor in the Molecular Torus

To calculate the expected seed factor of the molecular torus, we need an estimate of its energy density and characteristic photon energy. In the molecular torus, reverberation mapping [e.g., 3, 4] and near-infrared interferometric studies [5] of radio-quiet sources find that the inner radius of the molecular torus scales as $L_{d,45}^{1/2}$ (where $L_{d,45}$ is the accretion disk luminosity in units of 10^{45} erg s^{-1}). We assume this relation holds for radio-loud AGN. This relation implies that the molecular torus energy density is constant with both molecular torus size and luminosity. The best-fit relation and uncertainties on the scaling factor were found using the data in [3] and uncertainties were calculated using Wilk's Theorem. For the molecular torus we find $R_{\text{MT}} = 4.83 \pm 0.23 \times 10^{18} L_{d,45}^{1/2}$. The fraction of L_d which is reprocessed by the molecular torus (the covering factor) is $\xi_{\text{MT}} \sim 0.2$ [6, 7, 8]. We estimate the uncertainty on the covering factor as 50% of the expected value. This is justified by the distribution of molecular torus covering factors for high-z, luminous quasars found in [8]. We therefore estimate the molecular torus covering factor as $\xi_{\text{MT}} = 0.2 \pm 0.1$. The energy density of the molecular torus is therefore $U_{0,\text{MT}} = 2.28 \pm 1.16 \times 10^{-5}$ erg cm^{-3}. The molecular torus spectrum can be approximated using a blackbody spectrum with a temperature of $T = 1200$ K [9]. This gives a characteristic photon energy for the molecular torus of $\epsilon_{0,\text{MT}} = 5.7 \times 10^{-7}$, in units of electron rest mass energy. These quantities imply a molecular torus seed factor of $\text{SF}_{\text{MT}} = 3.92 \pm 0.11$.

1.3 Estimating the Seed Factor in the Broad-Line Region

Reverberation mapping of radio-quiet active galactic nuclei finds that the radius of the broad-line region is $R_{\text{BLR}} = 2.76 \pm 0.20 \times 10^{17} L_{d,45}^{1/2}$ [10]. We assume that this holds for radio-loud sources. The covering factor of the broad-line region is $\xi_{\text{BLR}} \sim 0.1$ [11]. Because the molecular torus is expected to be the same material as the broad-line region, but beyond the sublimation radius [12], the uncertainty in the broad-line region covering factor should be approximately the same as for the molecular torus. We therefore assume a 50% uncertainty in the broad-line region covering factor. The broad-line region covering factor is therefore estimated as $\xi_{\text{BLR}} = 0.1 \pm 0.05$. With this scaling relation and covering factor, the energy density of the broad-line region is $U_{0,\text{BLR}} = 3.49 \pm 1.80 \times 10^{-3}$ erg cm^{-3}. The broad-line region SED can be approximated by a blackbody with peak frequency of $\nu_0 = 1.5\nu_{\text{Ly}_\alpha}$ [1]. The characteristic photon energy of the broad-line region is therefore $\epsilon_{0,\text{BLR}} = 3 \times 10^{-5}$, in units of electron rest mass energy. These assumptions imply a broad-line region seed factor of $\text{SF}_{\text{BLR}} = 3.29 \pm 0.11$.

1.4 Description of SED Samples

Plots of the SEDs used and their fits are included in Supplementary Fig. 1-5.

1.4.1 LBAS Sample

The LBAS is the Fermi LAT Bright AGN Sample. A subsample of 48 quasi-simultaneous, multiwavelength SEDs for sources in the LBAS were presented in [13], selected from the LBAS solely on availability of Swift observations carried out between 2008 May and 2009 January. This selection criterion implies that the LBAS subsample should be relatively unbiased in terms of selection effects. The distribution of redshifts, optical, X-ray, and gamma-ray fluxes of the LBAS Sample are consistent with being the same as for the full LBAS [13]. Data for this sample is available via public repository at Vizier [14]. However, due to evident data missing in the tables available at Vizier, all data was extracted from plots in [13] using Dexter [15].

1.4.2 Giommi Sample

Part of the Giommi Sample is a sample of 105 quasi-simultaneous, multiwavelength SEDs belonging to 3 flux limited samples based on Planck, Swift, and Fermi data respectively as presented in [16]. The other part of the Giommi Sample is taken from a companion paper from the Planck Collaboration [17] which included 28 quasi-simultaneous SEDs which were not represented in [16] (i.e., not overlapping with the sample in [16]). Due to the companion nature of the Planck Collaboration paper, we have decided to refer to this collection of 2 samples as a single sample (we do, however, include the data for the Planck Collaboration paper in its own table). Swift-UVOT and *Fermi*-LAT data from [16] was accessed via public repository at Vizier [18]. Due to evident inconsistencies found in the tables available at Vizier, radio, ground-based optical, and Swift-XRT data from [16] was extracted from plots in the cited paper using Dexter [15]. All data from [17] was extracted from plots in the cited paper using Dexter [15].

1.4.3 DSSB Sample

The DSSB Sample is the sample of SEDs in [19]. DSSB stands for Dynamic SEDs of Southern Blazars. This is a sample of SEDs of the 22 gamma-ray brightest sources (according to the Fermi-LAT Third Source Catalog; [20]) observed in the Tracking Active Galactic Nuclei with Austral Milliarcsecond Interferometry program (TANAMI; [21]). Time periods of steady activity were identified in [19] by using Bayesian blocks to determine periods of constant gamma-ray flux, being subdivided further when such periods were longer than a year. [19] then searched for observations in other wavebands which were quasi-simultaneous to these identified time-periods. Data for this sample was accessed via public repository at Vizier [22].

We found that the DSSB Sample was needed to include enough BL Lacertae object SEDs for our testing against weak extragalactic jets as described in Section 1.6 of the Supplementary Information. The DSSB contributes somewhat minimally to our sample of FSRQs (only 11 FSRQ SEDs from the DSSB Sample are included in our final sample of SEDs).

1.4.4 Literature Search Sample

The Literature Search Sample is a sample of quasi-simultaneous, multiwavelength SEDs taken from the literature. Initially 7 sources were included in this sample: 3C 279, 3C 454.3, 3C 345, PKS 1510-089, PKS 0528+134, PKS 0420-015, and 1502+106. SEDs were excluded from the final sample based only on simultaneity, wavelength coverage, and steady-statedness. This left only SEDs from 3C 279 and 3C 454.3 in the sample. The SEDs included in the Literature Search Sample for these two sources (for completeness, including those which did not pass the cut on peak Compton frequency) are in [23, 24, 25, 26, 27, 28, 29]. All data in this sample was extracted from either tables or plots in the cited papers. Data was extracted from plots, using Dexter [15], in cases of evident inconsistencies in tables, missing data in tables, or in cases for which tables were not provided.

1.5 Impact of Variability in SED Samples

LBAS has a γ-ray integration time of 88 days for all SEDs. A companion paper [30] investigated the weekly lightcurves of the LBAS sources (note that 2 of the sources in our paper are missing in their data) in a 47 week period starting at the start of the LBAS period. We have calculated the normalized excess variance ($F_{var} = \sqrt{(S^2 - \langle\sigma_F^2\rangle)}/\langle F\rangle$) for the 13 weeks overlapping with the LBAS period, the normalized excess variance for the total 47 weeks, and compared these two quantities by calculating the fractional difference between them. This is detailed in the table below. Of note are (1) the normalized excess variance for all sources is below 1, with a maximum of about 0.8, and a median of about 0.4 (2) the fractional difference is only negative (i.e., the LBAS period has a larger excess variance than the total 47 weeks) for one source, and the median of the fractional difference is about 0.3, implying that the excess variance is generally a sizeable fraction smaller during the LBAS period than over the entire 47 week period (i.e., there is less variability in the integration time of the LBAS than these sources experience on average).

The DSSB SEDs were created via a Bayesian block method based on Fermi-LAT lightcurves to select periods of relatively steady flux.

The other samples were integrated over similarly short lengths of time, and thus it is likely that variability is similarly small and/or averaged out.

1.6 Comparison with Seed Factor of Weak Extragalactic Jets

To test that the seed factor distribution for powerful extragalactic jets is different from sources in which there is no significant external photon field for external Compton scattering we calculate the seed factor for weak extragalactic jets.

Beamed weak extragalactic jets generally correspond to BL Lacertae objects (BL Lacs, or BLLs). We have calculated the seed factor for sources in the LBAS Sample, Giommi Sample, and the DSSB Sample which are classified (in their respective papers) as BL Lacertae objects (see Supplementary Fig. 6, and compare with Fig. 1). The number of BL Lac SEDs which we could reliably fit is 21. This is about a third the number of FSRQ SEDs which we were able to reliably fit (62 SEDs for the FSRQs).

Supplementary Table 1: LBAS Normalized Excess Variances

0FGL Name	LBAS Time EV	Total EV	Fractional Difference of EV
J0457.1-2325	0.23	0.48	0.53
J1512.7-0905	0.80	0.95	0.15
J1256.1-0547	0.15	0.78	0.81
J2143.2+1741	0.37	0.51	0.27
J0349.8-2102	0.27	0.71	0.61
J1457.6-3538	0.58	0.60	0.04
J1229.1+0202	0.51	0.61	0.17
J2254.0+1609	0.35	0.92	0.62
J1522.2+3143	0.26	0.30	0.16
J0531.0+1331	0.43	0.81	0.47
J1504.4+1030	0.63	0.40	-0.57

The Kolmogorov-Smirnov test is not very sensitive to effects in the tails of a distribution. Global effects and the small sample size of the BL Lac sample reduce the reliability of the Kolmogorov-Smirnov test. We therefore applied the Anderson-Darling test and the Shapiro-Wilk test to large (10^7 variates) bootstrapped samples of the BL Lac and FSRQ seed factor distributions. Using a rejection significance threshold of 2σ, the BL Lac distribution is significantly rejected as normal by both of these tests ($> 2.58\sigma$ (p-value less than 0.01) using the Anderson-Darling test, and 3.55σ (p-value 0.00) using the Shapiro-Wilk test). The FSRQ distribution is not significantly rejected as normal by either of these tests ($1.64 - 1.96\sigma$ ($0.05 <$ p-value < 0.1) using the Anderson-Darling test, and 1.99σ (p-value 0.05) using the Shapiro-Wilk test). These results imply that the BL Lacs and FSRQs have distinct seed factor distributions, and thus that the BL Lacs and FSRQs likely emit gamma-rays through different processes.

A complicating factor is that BL Lacertae populations are known to be contaminated with "fake BL Lacs" [31], which are FSRQs which have been classified as BL Lacs due to their continuum emission outshining their broad-line emission. However, any contaminating FSRQs in our BL Lac sample are expected to have seed factors which behave the same as for the FSRQ sample (which is not a contaminated sample). Therefore, any differences between the FSRQ and BL Lac seed factor distributions are attributable to the real BL Lacs in the BL Lac sample.

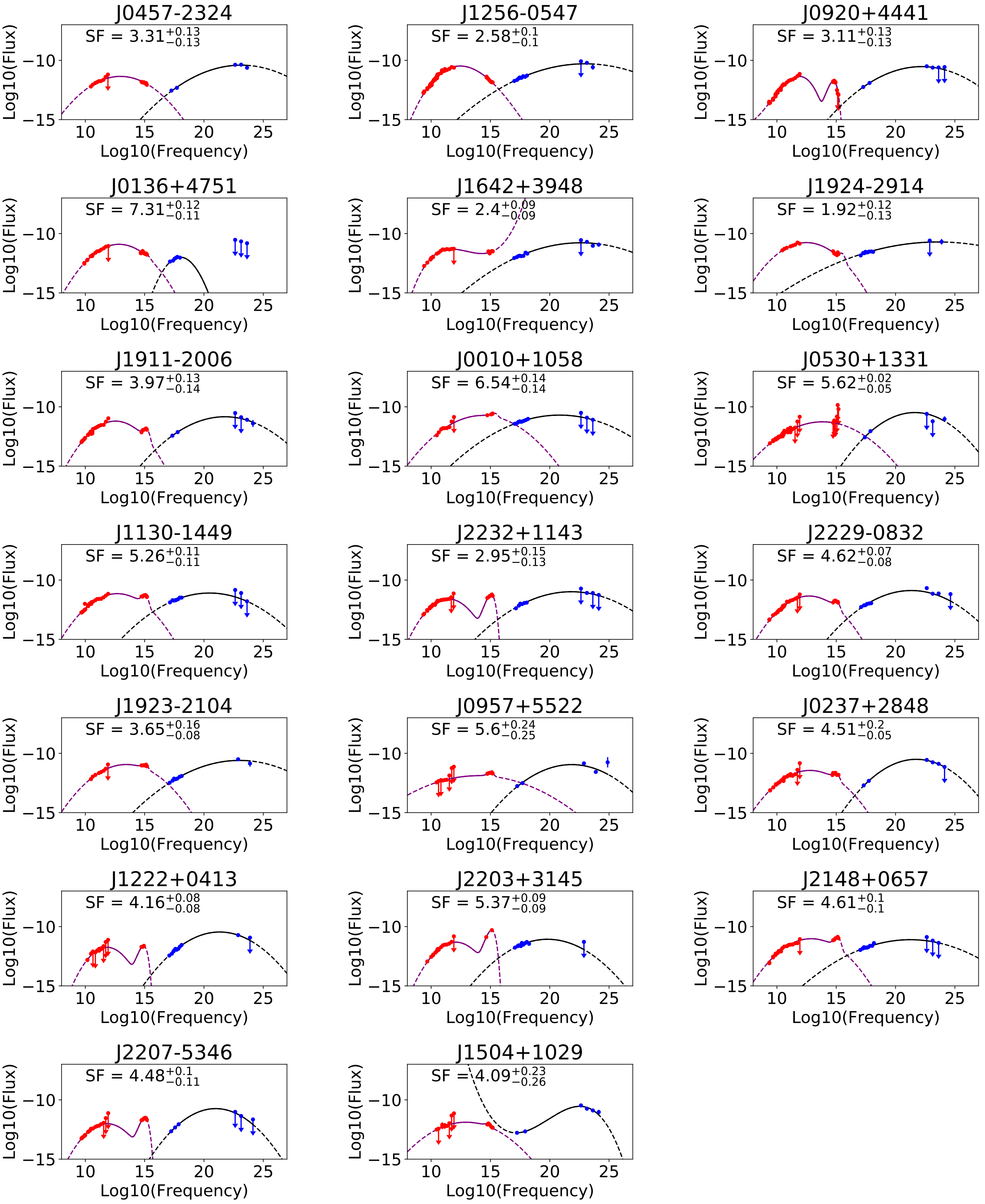

Supplementary Fig. 1: **Giommi Sample FSRQ SEDs.** The SEDs for the FSRQs in the Giommi Sample which passed all selection criteria. Red data points represent data we identified as being attributable to the synchrotron peak, big blue bump emission, or extended emission. Blue data represent data we identified as being primarily attributable to inverse Compton emission. The green curve shows the fit to the red data; the solid parts show the portion within the range of the red data, while the dashed parts show the extension of the fit beyond the range of the red data. The black curve represents the same, but for the blue data. Frequencies are in Hertz. Fluxes are in erg cm^{-2} s^{-1}. Error bars are as they were presented by the original authors. Downward pointing arrows represent upper limits as they were presented by the original authors.

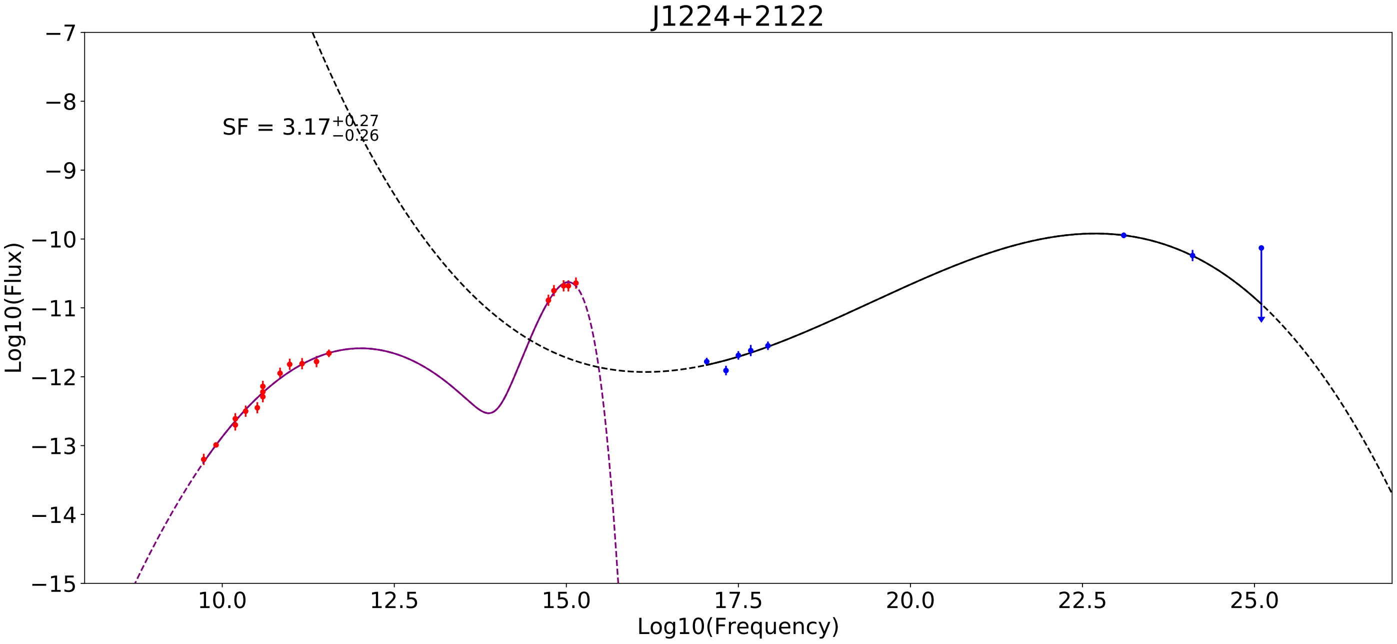

Supplementary Fig. 2: **Planck Sample FSRQ SEDs.** The SEDs for the FSRQs in the Planck Sample which passed all selection criteria. Red data points represent data we identified as being attributable to the synchrotron peak, big blue bump emission, or extended emission. Blue data represent data we identified as being primarily attributable to inverse Compton emission. The green curve shows the fit to the red data; the solid parts show the portion within the range of the red data, while the dashed parts show the extension of the fit beyond the range of the red data. The black curve represents the same, but for the blue data. Frequencies are in Hertz. Fluxes are in erg cm^{-2} s^{-1}. Error bars are as they were presented by the original authors. Downward pointing arrows represent upper limits as they were presented by the original authors.

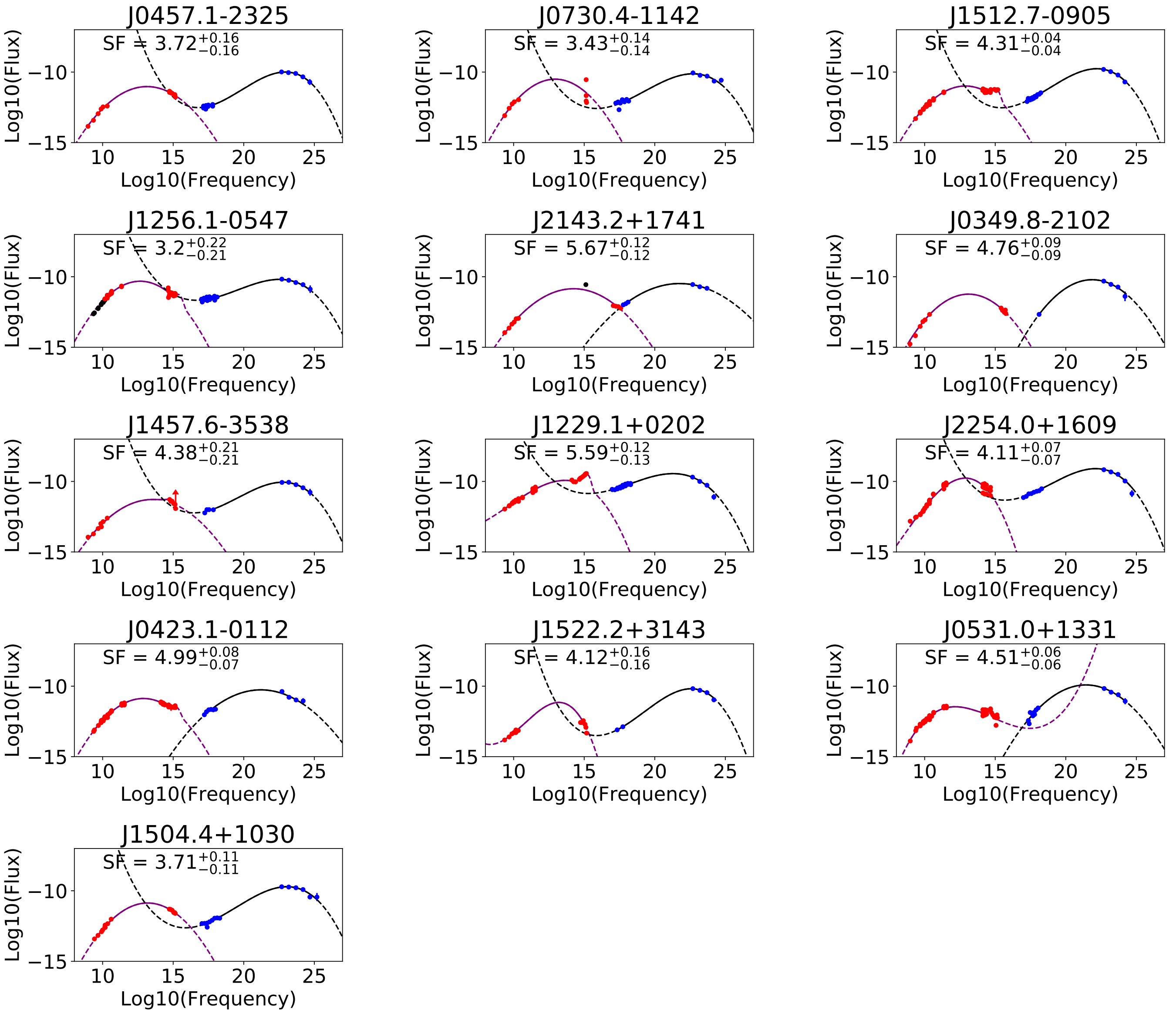

Supplementary Fig. 3: **LBAS Sample FSRQ SEDs.** The SEDs for the FSRQs in the LBAS Sample which passed all selection criteria. Red data points represent data we identified as being attributable to the synchrotron peak, big blue bump emission, or extended emission. Blue data represent data we identified as being primarily attributable to inverse Compton emission. The green curve shows the fit to the red data; the solid parts show the portion within the range of the red data, while the dashed parts show the extension of the fit beyond the range of the red data. The black curve represents the same, but for the blue data. Frequencies are in Hertz. Fluxes are in erg cm^{-2} s^{-1}. Error bars are as they were presented by the original authors. Downward pointing arrows represent upper limits as they were presented by the original authors.

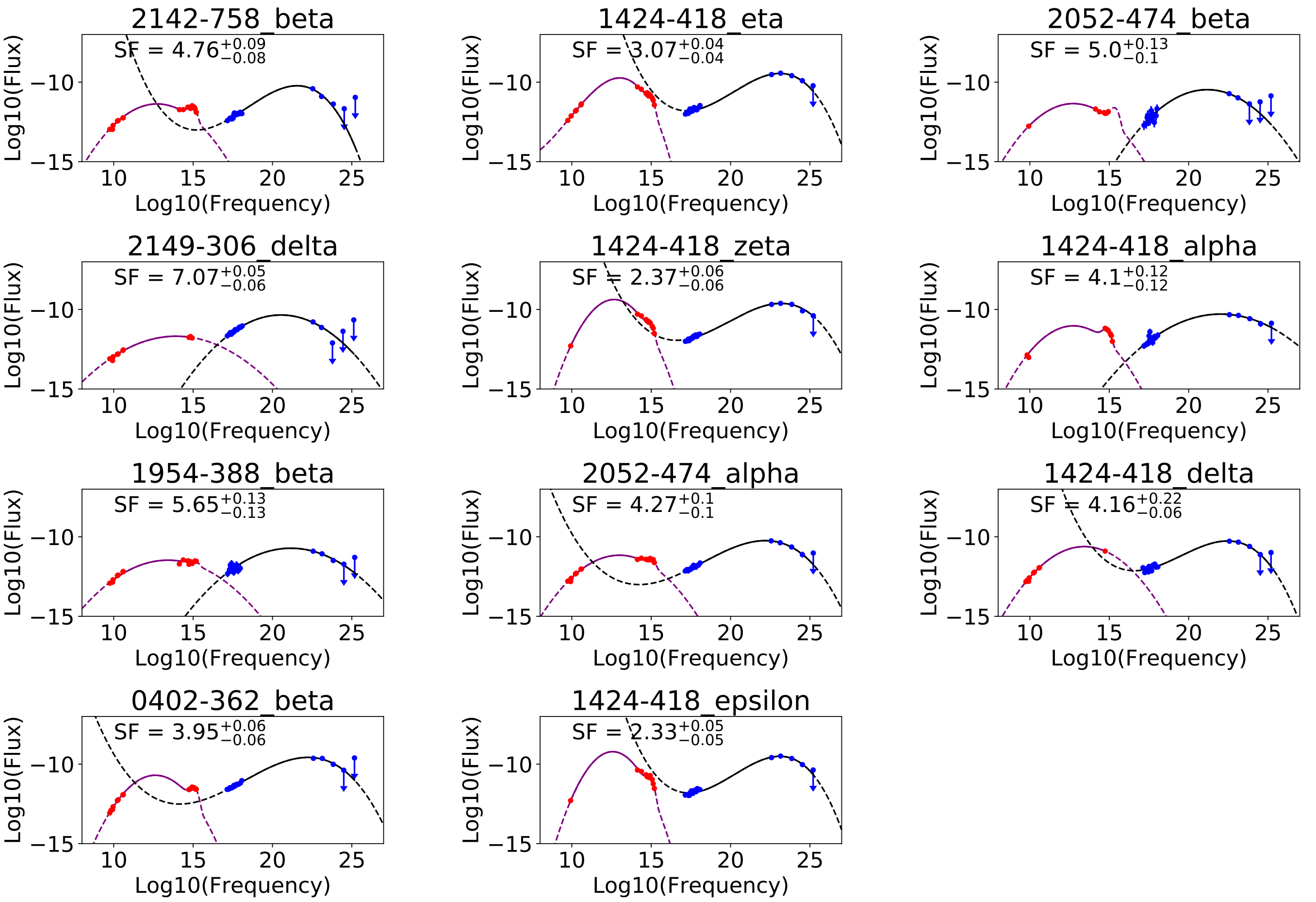

Supplementary Fig. 4: **DSSB Sample FSRQ SEDs.** The SEDs for the FSRQs in the DSSB Sample which passed all selection criteria. Red data points represent data we identified as being attributable to the synchrotron peak, big blue bump emission, or extended emission. Blue data represent data we identified as being primarily attributable to inverse Compton emission. The green curve shows the fit to the red data; the solid parts show the portion within the range of the red data, while the dashed parts show the extension of the fit beyond the range of the red data. The black curve represents the same, but for the blue data. Frequencies are in Hertz. Fluxes are in erg cm^{-2} s^{-1}. Error bars are as they were presented by the original authors. Upward/downward pointing arrows represent lower/upper limits as they were presented by the original authors.

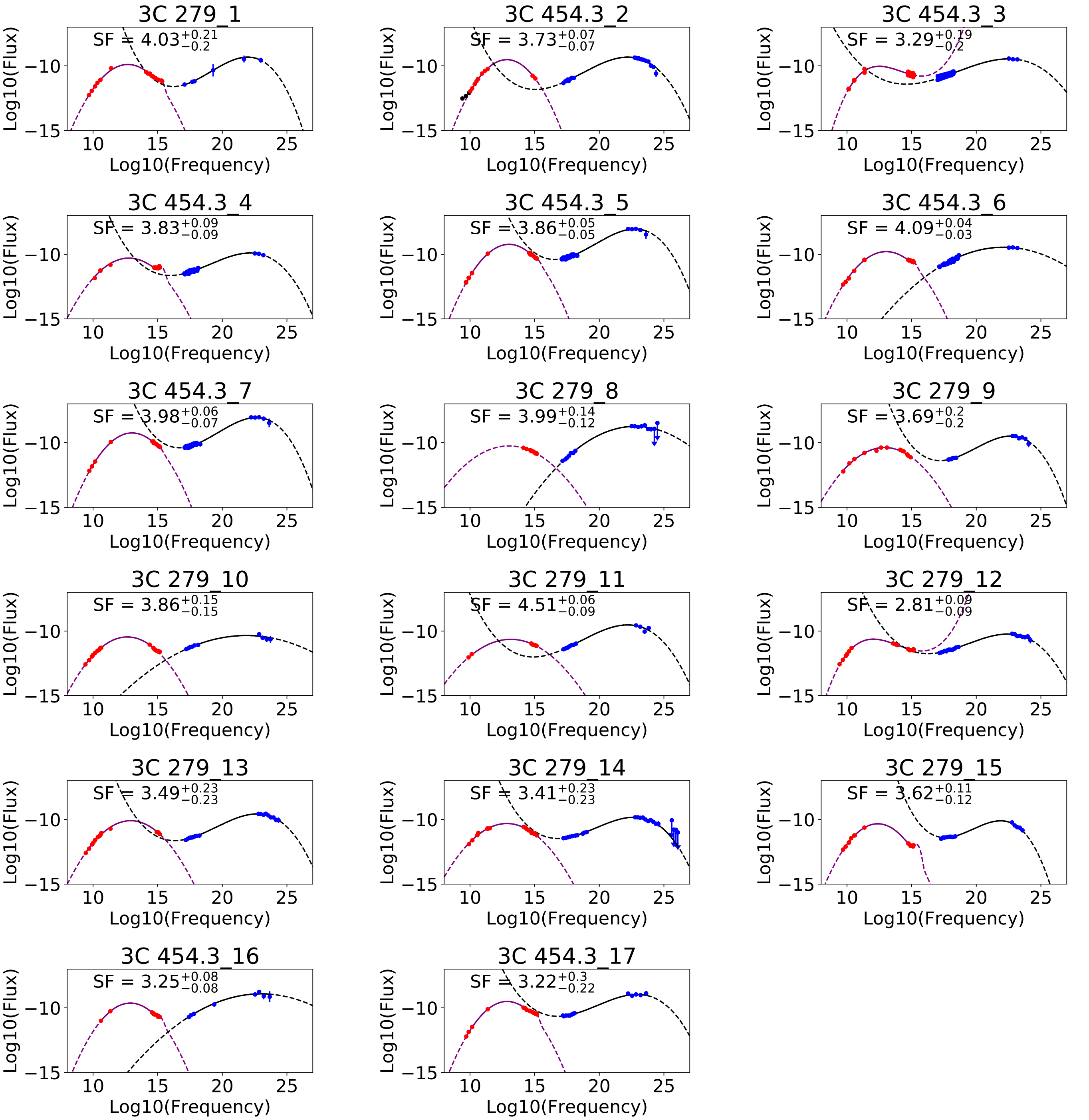

Supplementary Fig. 5: **LSS Sample FSRQ SEDs.** The SEDs for the FSRQs in the LSS Sample which passed all selection criteria. Red data points represent data we identified as being attributable to the synchrotron peak, big blue bump emission, or extended emission. Blue data represent data we identified as being primarily attributable to inverse Compton emission. The green curve shows the fit to the red data; the solid parts show the portion within the range of the red data, while the dashed parts show the extension of the fit beyond the range of the red data. The black curve represents the same, but for the blue data. Frequencies are in Hertz. Fluxes are in erg cm^{-2} s^{-1}. Error bars are as they were presented by the original authors. Naming is contracted to include only the source name and final trailing number as given in Supplementary Table 6. Downward pointing arrows represent upper limits as they were presented by the original authors.

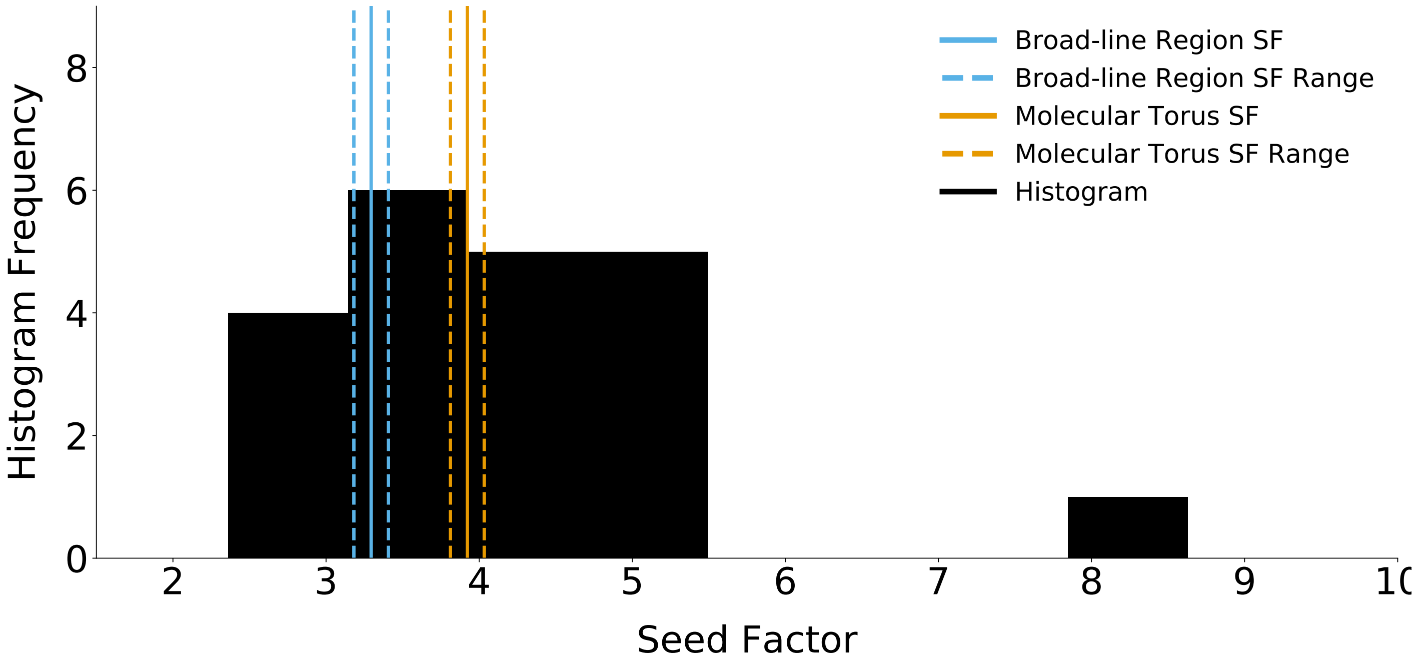

Supplementary Fig. 6: **Histogram of BL Lac seed factors.** A histogram showing the distribution of seed factors for our sample of BL Lacertae objects. We do not include a kernel density estimate here due to the small size of the BL Lac sample. For reference, the expected seed factor value and 1σ confidence interval for the broad-line region (blue) and molecular torus (orange) for FSRQs are shown by solid and dashed lines, respectively. See Sections 1.2 and 1.3 of the Supplementary Information for information on calculation of the plotted confidence intervals. The distribution of seed factors appears flat for BL Lacs as compared to for FSRQs. Compared with Fig. 1, it is clear that the FSRQ seed factor distribution has a sharper peak than the BL Lac seed factor distribution.

1.7 Seed Factor Tables

Supplementary Table 2: DSSB FSRQ Table

Source	SED Name	z	SF	Log10(ν_s)	Log10($\nu_s F_{\nu_s}$)	Log10(ν_{IC})	Log10($\nu_{IC} F_{\nu_{IC}}$)
2142-758	2142-758_beta	1.14	$4.76^{+0.09}_{-0.08}$	$12.73^{+0.01}_{-0.01}$	$-11.37^{+0.01}_{-0.01}$	$21.55^{+0.08}_{-0.08}$	$-10.22^{+0.05}_{-0.05}$
1424-418	1424-418_eta	1.52	$3.07^{+0.04}_{-0.04}$	$13.02^{+0.02}_{-0.02}$	$-9.73^{+0.02}_{-0.02}$	$23.10^{+0.04}_{-0.04}$	$-9.45^{+0.01}_{-0.01}$
2052-474	2052-474_beta	1.49	$5.00^{+0.13}_{-0.10}$	$12.73^{+0.04}_{-0.04}$	$-11.35^{+0.03}_{-0.03}$	$21.17^{+0.09}_{-0.12}$	$-10.47^{+0.06}_{-0.07}$
2149-306	2149-306_delta	2.35	$7.07^{+0.05}_{-0.06}$	$13.93^{+0.02}_{-0.02}$	$-11.68^{+0.01}_{-0.01}$	$20.54^{+0.05}_{-0.05}$	$-10.34^{+0.03}_{-0.03}$
1424-418	1424-418_zeta	1.52	$2.37^{+0.06}_{-0.06}$	$12.66^{+0.01}_{-0.01}$	$-9.37^{+0.02}_{-0.02}$	$23.18^{+0.05}_{-0.05}$	$-9.61^{+0.01}_{-0.01}$
1424-418	1424-418_alpha	1.52	$4.10^{+0.12}_{-0.12}$	$12.74^{+0.01}_{-0.01}$	$-11.03^{+0.01}_{-0.01}$	$22.02^{+0.12}_{-0.12}$	$-10.29^{+0.04}_{-0.04}$
1954-388	1954-388_beta	0.63	$5.65^{+0.13}_{-0.13}$	$13.42^{+0.04}_{-0.04}$	$-11.47^{+0.02}_{-0.02}$	$21.16^{+0.11}_{-0.12}$	$-10.72^{+0.06}_{-0.06}$
2052-474	2052-474_alpha	1.49	$4.27^{+0.10}_{-0.10}$	$12.99^{+0.03}_{-0.03}$	$-11.16^{+0.02}_{-0.02}$	$22.19^{+0.09}_{-0.10}$	$-10.24^{+0.07}_{-0.08}$
1424-418	1424-418_delta	1.52	$4.16^{+0.22}_{-0.06}$	$13.42^{+0.00}_{-0.00}$	$-10.61^{+0.00}_{-0.00}$	$22.45^{+0.05}_{-0.22}$	$-10.26^{+0.01}_{-0.07}$
0402-362	0402-362_beta	1.42	$3.95^{+0.06}_{-0.06}$	$12.62^{+0.01}_{-0.01}$	$-10.70^{+0.02}_{-0.02}$	$22.24^{+0.06}_{-0.06}$	$-9.57^{+0.04}_{-0.04}$
1424-418	1424-418_epsilon	1.52	$2.33^{+0.05}_{-0.05}$	$12.57^{+0.02}_{-0.02}$	$-9.21^{+0.04}_{-0.04}$	$23.10^{+0.04}_{-0.04}$	$-9.51^{+0.01}_{-0.01}$

Supplementary Table 3: LBAS FSRQ Table

Source	SED Name	z	SF	Log10(ν_s)	Log10($\nu_s F_{\nu_s}$)	Log10(ν_{IC})	Log10($\nu_{IC} F_{\nu_{IC}}$)
J0457.1-2325	J0457.1-2325	1.00	$3.72^{+0.16}_{-0.16}$	$13.14^{+0.02}_{-0.02}$	$-11.03^{+0.02}_{-0.02}$	$22.95^{+0.15}_{-0.15}$	$-10.00^{+0.04}_{-0.04}$
J0730.4-1142	J0730.4-1142	1.59	$3.43^{+0.14}_{-0.14}$	$12.94^{+0.02}_{-0.02}$	$-10.50^{+0.02}_{-0.02}$	$22.71^{+0.14}_{-0.14}$	$-10.12^{+0.05}_{-0.06}$
J1512.7-0905	J1512.7-0905	0.36	$4.31^{+0.04}_{-0.04}$	$12.89^{+0.01}_{-0.01}$	$-10.99^{+0.01}_{-0.01}$	$22.20^{+0.03}_{-0.03}$	$-9.76^{+0.02}_{-0.02}$
J1256.1-0547	J1256.1-0547	0.54	$3.20^{+0.22}_{-0.21}$	$12.67^{+0.02}_{-0.02}$	$-10.32^{+0.02}_{-0.02}$	$22.53^{+0.20}_{-0.21}$	$-10.19^{+0.08}_{-0.07}$
J2143.2+1741	J2143.2+1741	0.21	$5.67^{+0.12}_{-0.12}$	$14.25^{+0.02}_{-0.02}$	$-10.85^{+0.02}_{-0.02}$	$21.77^{+0.11}_{-0.12}$	$-10.48^{+0.05}_{-0.05}$
J0349.8-2102	J0349.8-2102	2.94	$4.76^{+0.09}_{-0.09}$	$13.12^{+0.02}_{-0.02}$	$-11.23^{+0.02}_{-0.02}$	$21.87^{+0.09}_{-0.09}$	$-10.21^{+0.05}_{-0.05}$
J1457.6-3538	J1457.6-3538	1.42	$4.38^{+0.21}_{-0.21}$	$13.52^{+0.03}_{-0.03}$	$-11.28^{+0.02}_{-0.03}$	$22.76^{+0.21}_{-0.20}$	$-10.06^{+0.08}_{-0.08}$
J1229.1+0202	J1229.1+0202	0.16	$5.59^{+0.12}_{-0.13}$	$13.63^{+0.09}_{-0.10}$	$-9.93^{+0.04}_{-0.04}$	$21.29^{+0.09}_{-0.08}$	$-9.45^{+0.04}_{-0.04}$
J2254.0+1609	J2254.0+1609	0.86	$4.11^{+0.07}_{-0.07}$	$12.88^{+0.02}_{-0.02}$	$-9.77^{+0.02}_{-0.02}$	$22.11^{+0.10}_{-0.07}$	$-9.09^{+0.04}_{-0.05}$
J0423.1-0112	J0423.1-0112	0.92	$4.99^{+0.08}_{-0.07}$	$12.89^{+0.01}_{-0.01}$	$-10.88^{+0.01}_{-0.01}$	$21.22^{+0.07}_{-0.07}$	$-10.26^{+0.04}_{-0.04}$
J1522.2+3143	J1522.2+3143	1.49	$4.12^{+0.16}_{-0.16}$	$13.21^{+0.04}_{-0.04}$	$-11.15^{+0.07}_{-0.06}$	$22.58^{+0.15}_{-0.14}$	$-10.19^{+0.06}_{-0.06}$
J0531.0+1331	J0531.0+1331	2.07	$4.51^{+0.06}_{-0.06}$	$12.17^{+0.03}_{-0.03}$	$-11.46^{+0.02}_{-0.02}$	$21.43^{+0.05}_{-0.04}$	$-9.92^{+0.04}_{-0.04}$
J1504.4+1030	J1504.4+1030	1.84	$3.71^{+0.11}_{-0.11}$	$13.19^{+0.01}_{-0.01}$	$-9.71^{+0.04}_{-0.04}$	$23.06^{+0.11}_{-0.11}$	$-9.71^{+0.04}_{-0.04}$

Supplementary Table 4: Giommi FSRQ Table

Source	SED Name	z	SF	Log10(ν_s)	Log10($\nu_s F_{\nu_s}$)	Log10(ν_{IC})	Log10($\nu_{IC} F_{\nu_{IC}}$)
J0457-2324	J0457-2324	1.00	$3.31^{+0.13}_{-0.13}$	$12.97^{+0.04}_{-0.04}$	$-11.35^{+0.02}_{-0.02}$	$23.14^{+0.12}_{-0.12}$	$-10.41^{+0.07}_{-0.07}$
J1256-0547	J1256-0547	0.54	$2.58^{+0.10}_{-0.10}$	$12.45^{+0.02}_{-0.02}$	$-10.48^{+0.02}_{-0.02}$	$22.97^{+0.09}_{-0.09}$	$-10.29^{+0.04}_{-0.04}$
J0920+4441	J0920+4441	2.19	$3.11^{+0.13}_{-0.13}$	$11.89^{+0.03}_{-0.03}$	$-11.35^{+0.04}_{-0.04}$	$22.21^{+0.12}_{-0.12}$	$-10.53^{+0.07}_{-0.07}$
J0136+4751	J0136+4751	0.86	$7.31^{+0.12}_{-0.11}$	$12.85^{+0.03}_{-0.02}$	$-10.90^{+0.01}_{-0.03}$	$18.01^{+0.10}_{-0.11}$	$-11.99^{+0.05}_{-0.06}$
J1642+3948	J1642+3948	0.59	$2.40^{+0.09}_{-0.09}$	$11.73^{+0.03}_{-0.04}$	$-11.31^{+0.02}_{-0.02}$	$22.60^{+0.08}_{-0.08}$	$-10.78^{+0.03}_{-0.03}$
J1924-2914	J1924-2914	0.35	$1.92^{+0.12}_{-0.13}$	$12.48^{+0.05}_{-0.05}$	$-10.76^{+0.03}_{-0.03}$	$23.59^{+0.11}_{-0.11}$	$-10.71^{+0.03}_{-0.03}$
J1911-2006	J1911-2006	1.12	$3.97^{+0.13}_{-0.14}$	$12.54^{+0.04}_{-0.04}$	$-11.20^{+0.03}_{-0.03}$	$21.76^{+0.13}_{-0.12}$	$-10.84^{+0.08}_{-0.09}$
J0010+1058	J0010+1058	0.09	$6.54^{+0.14}_{-0.14}$	$14.36^{+0.07}_{-0.07}$	$-10.73^{+0.02}_{-0.02}$	$20.84^{+0.11}_{-0.11}$	$-10.70^{+0.04}_{-0.04}$
J0530+1331	J0530+1331	2.07	$5.62^{+0.02}_{-0.05}$	$13.85^{+0.00}_{-0.03}$	$-11.26^{+0.00}_{-0.02}$	$21.63^{+0.00}_{-0.07}$	$-10.48^{+0.00}_{-0.06}$
J1130-1449	J1130-1449	1.18	$5.26^{+0.11}_{-0.11}$	$12.69^{+0.04}_{-0.04}$	$-11.14^{+0.03}_{-0.03}$	$20.47^{+0.10}_{-0.10}$	$-11.09^{+0.04}_{-0.04}$
J2232+1143	J2232+1143	1.04	$2.95^{+0.15}_{-0.13}$	$11.46^{+0.04}_{-0.05}$	$-11.59^{+0.03}_{-0.03}$	$21.82^{+0.11}_{-0.14}$	$-10.98^{+0.05}_{-0.06}$
J2229-0832	J2229-0832	1.56	$4.62^{+0.07}_{-0.08}$	$12.71^{+0.04}_{-0.04}$	$-11.34^{+0.04}_{-0.04}$	$21.33^{+0.06}_{-0.06}$	$-10.88^{+0.02}_{-0.02}$
J1923-2104	J1923-2104	0.87	$3.65^{+0.16}_{-0.08}$	$13.46^{+0.06}_{-0.06}$	$-10.94^{+0.03}_{-0.03}$	$22.99^{+0.07}_{-0.12}$	$-10.59^{+0.07}_{-0.04}$
J0957+5522	J0957+5522	0.90	$5.60^{+0.24}_{-0.25}$	$13.97^{+0.26}_{-0.20}$	$-11.88^{+0.04}_{-0.04}$	$21.85^{+0.13}_{-0.14}$	$-10.95^{+0.09}_{-0.09}$
J0237+2848	J0237+2848	1.21	$4.51^{+0.20}_{-0.05}$	$12.78^{+0.05}_{-0.05}$	$-11.44^{+0.03}_{-0.03}$	$21.75^{+0.00}_{-0.12}$	$-10.50^{+0.00}_{-0.06}$
J1222+0413	J1222+0413	0.97	$4.16^{+0.08}_{-0.08}$	$11.83^{+0.05}_{-0.05}$	$-11.74^{+0.04}_{-0.04}$	$21.33^{+0.06}_{-0.06}$	$-10.45^{+0.05}_{-0.05}$
J2203+3145	J2203+3145	0.29	$5.37^{+0.09}_{-0.09}$	$12.07^{+0.03}_{-0.03}$	$-11.31^{+0.03}_{-0.03}$	$19.82^{+0.07}_{-0.08}$	$-11.07^{+0.03}_{-0.03}$
J2148+0657	J2148+0657	1.00	$4.61^{+0.10}_{-0.10}$	$12.89^{+0.03}_{-0.03}$	$-11.02^{+0.02}_{-0.02}$	$21.25^{+0.09}_{-0.09}$	$-11.10^{+0.03}_{-0.03}$
J2207-5346	J2207-5346	1.22	$4.48^{+0.10}_{-0.11}$	$11.83^{+0.05}_{-0.05}$	$-12.01^{+0.04}_{-0.04}$	$20.99^{+0.08}_{-0.08}$	$-10.74^{+0.08}_{-0.08}$
J1504+1029	J1504+1029	1.84	$4.09^{+0.23}_{-0.26}$	$12.94^{+0.09}_{-0.02}$	$-11.88^{+0.01}_{-0.04}$	$22.53^{+0.24}_{-0.23}$	$-10.55^{+0.09}_{-0.08}$

Supplementary Table 5: Planck FSRQ Table

Source	SED Name	z	SF	Log10(ν_s)	Log10($\nu_s F_{\nu_s}$)	Log10(ν_{IC})	Log10($\nu_{IC} F_{\nu_{IC}}$)
J1224+2122	J1224+2122	0.43	$3.17^{+0.27}_{-0.26}$	$12.01^{+0.02}_{-0.02}$	$-11.59^{+0.03}_{-0.03}$	$22.69^{+0.27}_{-0.26}$	$-9.92^{+0.10}_{-0.09}$

Supplementary Table 6: LSS FSRQ Table

Source	SED Name	z	SF	Log10(ν_s)	Log10($\nu_s F_{\nu_s}$)	Log10(ν_{IC})	Log10($\nu_{IC} F_{\nu_{IC}}$)
3C 279	3C_279_January_1 [23]	0.54	$4.03^{+0.21}_{-0.20}$	$12.70^{+0.01}_{-0.01}$	$-9.89^{+0.01}_{-0.01}$	$21.96^{+0.22}_{-0.19}$	$-9.32^{+0.10}_{-0.09}$
3C 454.3	3C_454.3_2 [24]	0.86	$3.73^{+0.07}_{-0.07}$	$12.85^{+0.02}_{-0.01}$	$-9.52^{+0.02}_{-0.02}$	$22.23^{+0.06}_{-0.06}$	$-9.32^{+0.04}_{-0.04}$
3C 454.3	3C_454.3_54673_54693_3 [25]	0.86	$3.29^{+0.19}_{-0.20}$	$12.50^{+0.03}_{-0.03}$	$-10.03^{+0.02}_{-0.01}$	$22.51^{+0.19}_{-0.19}$	$-9.46^{+0.08}_{-0.08}$
3C 454.3	3C_454.3_54800_54845_4 [25]	0.86	$3.83^{+0.09}_{-0.09}$	$12.77^{+0.02}_{-0.02}$	$-10.29^{+0.01}_{-0.02}$	$22.15^{+0.09}_{-0.09}$	$-9.90^{+0.03}_{-0.03}$
3C 454.3	3C_454.3_flare_5 [26]	0.86	$3.86^{+0.05}_{-0.05}$	$13.02^{+0.00}_{-0.00}$	$-9.23^{+0.01}_{-0.01}$	$22.76^{+0.04}_{-0.05}$	$-8.04^{+0.02}_{-0.02}$
3C 454.3	3C_454.3_54617_54618_6 [25]	0.86	$4.09^{+0.04}_{-0.03}$	$13.04^{+0.02}_{-0.02}$	$-9.79^{+0.02}_{-0.02}$	$22.12^{+0.02}_{-0.02}$	$-9.45^{+0.01}_{-0.01}$
3C 454.3	3C_454.3_post-flare_7 [26]	0.86	$3.98^{+0.06}_{-0.07}$	$13.02^{+0.01}_{-0.01}$	$-9.24^{+0.01}_{-0.01}$	$22.64^{+0.06}_{-0.06}$	$-8.05^{+0.02}_{-0.02}$
3C 279	3C_279_Period_D_8 [27]	0.54	$3.99^{+0.14}_{-0.12}$	$13.01^{+0.06}_{-0.05}$	$-10.25^{+0.02}_{-0.02}$	$22.79^{+0.04}_{-0.06}$	$-8.74^{+0.03}_{-0.04}$
3C 279	3C_279_Period_D_9 [28]	0.54	$3.69^{+0.20}_{-0.20}$	$12.91^{+0.05}_{-0.03}$	$-10.37^{+0.04}_{-0.02}$	$22.68^{+0.17}_{-0.13}$	$-9.48^{+0.08}_{-0.06}$
3C 279	3C_279_Period_F_10 [28]	0.54	$3.86^{+0.15}_{-0.15}$	$12.65^{+0.02}_{-0.01}$	$-10.45^{+0.01}_{-0.02}$	$21.85^{+0.10}_{-0.08}$	$-10.34^{+0.03}_{-0.04}$
3C 279	3C_279_Period_G_11 [28]	0.54	$4.51^{+0.06}_{-0.09}$	$13.15^{+0.04}_{-0.06}$	$-10.64^{+0.03}_{-0.02}$	$22.21^{+0.05}_{-0.05}$	$-9.52^{+0.02}_{-0.02}$
3C 279	3C_279_Period_A_12 [28]	0.54	$2.81^{+0.09}_{-0.09}$	$12.04^{+0.06}_{-0.05}$	$-10.62^{+0.05}_{-0.06}$	$22.43^{+0.07}_{-0.07}$	$-10.23^{+0.02}_{-0.02}$
3C 279	3C_279_Period_B_13 [28]	0.54	$3.49^{+0.23}_{-0.23}$	$12.91^{+0.03}_{-0.03}$	$-10.09^{+0.02}_{-0.03}$	$22.69^{+0.17}_{-0.17}$	$-9.57^{+0.10}_{-0.10}$
3C 279	3C_279_Period_C_14 [28]	0.54	$3.41^{+0.23}_{-0.23}$	$12.85^{+0.03}_{-0.03}$	$-10.31^{+0.02}_{-0.02}$	$22.70^{+0.19}_{-0.18}$	$-9.83^{+0.07}_{-0.06}$
3C 279	3C_279_Period_H_15 [28]	0.54	$3.62^{+0.11}_{-0.12}$	$12.38^{+0.02}_{-0.02}$	$-10.33^{+0.03}_{-0.02}$	$21.88^{+0.11}_{-0.11}$	$-10.10^{+0.09}_{-0.08}$
3C 454.3	3C_454.3_pre-flare_16 [29]	0.86	$3.25^{+0.08}_{-0.08}$	$12.89^{+0.03}_{-0.03}$	$-9.63^{+0.03}_{-0.03}$	$23.00^{+0.07}_{-0.07}$	$-8.91^{+0.04}_{-0.04}$
3C 454.3	3C_454.3_pre-flare_17 [26]	0.86	$3.22^{+0.30}_{-0.22}$	$12.87^{+0.02}_{-0.02}$	$-9.50^{+0.03}_{-0.02}$	$22.93^{+0.18}_{-0.31}$	$-8.96^{+0.07}_{-0.08}$

Supplementary Table 7: DSSB BL Lac Table

Source	SED Name	z	SF	Log10(ν_s)	Log10($\nu_s F_{\nu_s}$)	Log10(ν_{IC})	Log10($\nu_{IC} F_{\nu_{IC}}$)
0537-441	0537-441_alpha	0.89	$3.93^{+0.06}_{-0.06}$	$13.21^{+0.01}_{-0.01}$	$-10.61^{+0.01}_{-0.01}$	$22.48^{+0.06}_{-0.06}$	$-10.22^{+0.03}_{-0.03}$
0537-441	0537-441_zeta	0.89	$4.15^{+0.15}_{-0.16}$	$13.24^{+0.00}_{-0.00}$	$-10.70^{+0.00}_{-0.00}$	$22.24^{+0.16}_{-0.16}$	$-10.42^{+0.05}_{-0.05}$
0426-380	0426-380_epsilon	1.11	$3.55^{+0.08}_{-0.08}$	$13.61^{+0.02}_{-0.02}$	$-10.68^{+0.02}_{-0.02}$	$23.34^{+0.08}_{-0.08}$	$-10.13^{+0.02}_{-0.02}$
0537-441	0537-441_delta	0.89	$2.36^{+0.04}_{-0.04}$	$12.87^{+0.00}_{-0.00}$	$-9.81^{+0.00}_{-0.00}$	$23.50^{+0.04}_{-0.04}$	$-9.84^{+0.02}_{-0.02}$
0537-441	0537-441_epsilon	0.89	$3.22^{+0.23}_{-0.23}$	$13.44^{+0.00}_{-0.00}$	$-10.46^{+0.00}_{-0.00}$	$23.37^{+0.23}_{-0.22}$	$-10.15^{+0.08}_{-0.08}$
0426-380	0426-380_gamma	1.11	$3.55^{+0.28}_{-0.28}$	$13.47^{+0.02}_{-0.02}$	$-11.30^{+0.01}_{-0.01}$	$23.29^{+0.29}_{-0.28}$	$-10.60^{+0.06}_{-0.06}$
1057-797	1057-797_alpha	0.58	$4.71^{+0.19}_{-0.19}$	$13.08^{+0.00}_{-0.00}$	$-11.47^{+0.00}_{-0.00}$	$21.62^{+0.19}_{-0.19}$	$-10.98^{+0.06}_{-0.06}$
0447-439	0447-439_gamma	0.11	$3.89^{+0.20}_{-0.19}$	$15.02^{+0.05}_{-0.05}$	$-10.26^{+0.01}_{-0.01}$	$23.85^{+0.16}_{-0.19}$	$-10.81^{+0.05}_{-0.05}$
0537-441	0537-441_gamma	0.89	$4.05^{+0.08}_{-0.08}$	$13.36^{+0.02}_{-0.02}$	$-10.33^{+0.01}_{-0.01}$	$22.40^{+0.07}_{-0.07}$	$-10.16^{+0.03}_{-0.03}$
0537-441	0537-441_beta	0.89	$4.83^{+0.07}_{-0.07}$	$13.55^{+0.03}_{-0.03}$	$-11.12^{+0.01}_{-0.01}$	$22.13^{+0.10}_{-0.06}$	$-10.34^{+0.03}_{-0.03}$

Supplementary Table 8: LBAS BL Lac Table

Source	SED Name	z	SF	Log10(ν_s)	Log10($\nu_s F_{\nu_s}$)	Log10(ν_{IC})	Log10($\nu_{IC} F_{\nu_{IC}}$)
J1751.5+0935	J1751.5+0935	0.32	$2.99^{+0.25}_{-0.25}$	$12.87^{+0.01}_{-0.01}$	$-10.22^{+0.02}_{-0.02}$	$22.80^{+0.21}_{-0.24}$	$-10.39^{+0.07}_{-0.10}$
J0712.9+5034	J0712.9+5034	0.50	$3.10^{+0.18}_{-0.18}$	$13.55^{+0.04}_{-0.04}$	$-11.21^{+0.02}_{-0.02}$	$23.58^{+0.16}_{-0.17}$	$-10.98^{+0.08}_{-0.07}$
J0855.4+2009	J0855.4+2009	0.31	$4.81^{+0.09}_{-0.09}$	$13.47^{+0.01}_{-0.01}$	$-9.96^{+0.02}_{-0.02}$	$21.40^{+0.09}_{-0.09}$	$-10.50^{+0.04}_{-0.04}$
J0538.8-4403	J0538.8-4403	0.89	$3.80^{+0.19}_{-0.16}$	$13.27^{+0.03}_{-0.03}$	$-10.64^{+0.02}_{-0.02}$	$22.78^{+0.25}_{-0.17}$	$-10.05^{+0.10}_{-0.06}$

Supplementary Table 9: Giommi BL Lac Table

Source	SED Name	z	SF	Log10(ν_s)	Log10($\nu_s F_{\nu_s}$)	Log10(ν_{IC})	Log10($\nu_{IC} F_{\nu_{IC}}$)
J0738+1742	J0738+1742	0.42	$3.03^{+0.20}_{-0.20}$	$13.82^{+0.04}_{-0.04}$	$-10.99^{+0.01}_{-0.01}$	$23.75^{+0.22}_{-0.19}$	$-11.10^{+0.08}_{-0.09}$
J2202+4216	J2202+4216	0.07	$4.93^{+0.14}_{-0.14}$	$13.75^{+0.04}_{-0.04}$	$-10.29^{+0.02}_{-0.02}$	$21.73^{+0.13}_{-0.13}$	$-10.48^{+0.04}_{-0.04}$
J0538-4405	J0538-4405	0.89	$3.69^{+0.19}_{-0.20}$	$13.52^{+0.02}_{-0.02}$	$-10.27^{+0.01}_{-0.01}$	$23.01^{+0.19}_{-0.19}$	$-9.92^{+0.03}_{-0.03}$
J0818+4222	J0818+4222	0.53	$4.29^{+0.14}_{-0.14}$	$13.09^{+0.03}_{-0.03}$	$-11.45^{+0.02}_{-0.02}$	$22.06^{+0.12}_{-0.12}$	$-10.96^{+0.09}_{-0.09}$
J0550-3216	J0550-3216	0.07	$8.63^{+0.09}_{-0.08}$	$13.58^{+0.02}_{-0.02}$	$-10.87^{+0.02}_{-0.02}$	$18.11^{+0.07}_{-0.08}$	$-10.57^{+0.03}_{-0.03}$
J1800+7828	J1800+7828	0.68	$4.43^{+0.12}_{-0.12}$	$13.59^{+0.02}_{-0.02}$	$-10.82^{+0.01}_{-0.01}$	$22.10^{+0.12}_{-0.12}$	$-10.95^{+0.05}_{-0.05}$
J1517-2422	J1517-2422	0.05	$4.76^{+0.08}_{-0.08}$	$13.72^{+0.03}_{-0.03}$	$-10.34^{+0.02}_{-0.02}$	$21.79^{+0.08}_{-0.08}$	$-10.70^{+0.03}_{-0.03}$

2 Supplementary Methods

2.1 Empirical Model Fitting of SEDs

All SEDs were fit using the continuous simulated annealing global optimization algorithm [32] as implemented by Goffe, Ferrier, and Rodgers [33], version 3.2.

2.1.1 Models

We used a few different empirical models to fit the SEDs. These are a log-parabola, log-parabola with a thermal (big-blue bump) component, third-degree polynomial, third-degree polynomial with a thermal (big-blue bump) component, and a log-parabola with a low-energy power-law cutoff (to model extended emission as necessary in a very small number of SEDs). Choosing between these models was based on 3σ significance preference over the null-hypothesis model (i.e., via Wilk's theorem imposing a 3σ cutoff to reject the model with less degrees of freedom). Model choice only disobeyed this criteria in a very small number of cases where the null-hypothesis model clearly visually disagreed with the SED data (in almost all such cases the significance of rejecting the null-hypothesis model was nearly 3σ).

We list the mathematical expressions for our models below. Here the different models are listed as LP (Log-Parabola), Poly3D (third-degree polynomial), BBB (big-blue bump thermal spectrum), LPBBB (log-parabola with big-blue bump added), Poly3DBBB (third-degree polynomial with big-blue bump added), PL (power-law), and LPPL (log-parabola with low-energy power-law cutoff). In Poly3D ν_l is the base-10 logarithm of the frequency, and $\nu_{l,0}$ is the pivot logarithmic frequency. BBB and PL are defined alone only to aid in comprehension. These two models were never used alone.

$$\mathrm{LP}(\nu) = N_0 \left(\frac{\nu}{\nu_p}\right)^{-b\mathrm{Log10}(\nu/\nu_p)} \tag{10}$$

$$\mathrm{Poly3D}(\nu_l) = a(\nu_l - \nu_{l,0})^3 + b(\nu_l - \nu_{l,0})^2 + c(\nu_l - \nu_{l,0}) + d \tag{11}$$

$$\mathrm{BBB}(\nu) = \pi N_{\mathrm{BBB}} \frac{2h\left(\nu\left(1+z\right)\right)^4}{c^2} \left(\mathrm{Exp}\left[\frac{h\nu\left(1+z\right)}{k_B T_{\mathrm{BBB}}}\right] - 1\right)^{-1} \tag{12}$$

$$\mathrm{LPBBB}(\nu) = \mathrm{LP}(\nu) + \mathrm{BBB}(\nu) \tag{13}$$

$$\mathrm{Poly3DBBB}(\nu) = \mathrm{Poly3D}(\mathrm{Log10}(\nu)) + \mathrm{BBB}(\nu) \tag{14}$$

$$\mathrm{PL} = N_0 \left(\frac{\nu}{\nu_0}\right)^{-\alpha} \tag{15}$$

$$\mathrm{LPPL}(\nu) = \begin{cases} \mathrm{LP}(\nu), & \text{if } \nu >= \nu_0 \\ \mathrm{PL}(\nu), & \text{if } \nu < \nu_0 \end{cases} \tag{16}$$

We corrected data for attenuation due to the extragalactic background light using the Dominguez 2011 [34] model as implemented in `gammapy`.

2.2 Effective Coverage of SEDs

Well-sampled in the context of our paper was defined using spectral curvature. SEDs were excluded if a power-law was preferred over a log-parabola at the $>= 2\sigma$ level. We chose to use curvature as a proxy for "well-sampledness" since blazar spectral components are expected to have curvature. Therefore, if an SED cannot significantly constrain the curvature of either the synchrotron or IC component, the SED must not be well-sampled (either due to actual wavelength coverage or due to measurement uncertainties). A statistical test for what we term "effective coverage" is preferred over observer choice, since it reduces the possibility of any observer bias and places all SEDs on an equal statistical footing.

2.3 Bootstrapping Methods

2.3.1 Error-Modified Bootstrap.

To estimate uncertainties of quantities for which the likelihood is unknown or unreasonable to calculate or sample, uncertainties can be estimated via bootstrapping. Bootstrapping is defined by estimating a population distribution by creating a sample through the use of resampling with replacement. Key to this discussion is the idea of bootstrapping as a method of sampling an observed distribution. It is upon this idea that we create an extension of the bootstrap to incorporate the uncertainties estimated for the observed variates (that is the observed quantities in a sample; e.g., the observed seed factors).

The error-modified bootstrap extends the standard bootstrap by drawing, for each resampling of the observed variates, a random value of each individual resampled variate from its respective distribution as defined by its respective uncertainties. This creates a population distribution estimate which incorporates the measured sample uncertainties. This method also allows for an asymptotically continuous distribution of sample statistics (i.e., continuous in the limit of an infinite number of samples), as opposed to the necessarily discrete distribution produced by the standard bootstrap (since there is a finite number of combinations of observed variates in the case of the standard bootstrap).

2.3.2 Parameter Bootstrap.

For uncertainty estimation of values determined by a set of parameters (e.g., the peak frequency of a third-degree polynomial), it was necessary to perform a bootstrap on the measured parameters. This procedure is to create a random sample of parameters where each sample parameter, θ_i^*, is drawn from the distribution of $\hat{\theta}_i$, as defined by the uncertainties on $\hat{\theta}_i$. That is, a single sample in the bootstrap is a set $\{\theta_i^*\}$ such that each θ_i^* has been drawn from the distribution of $\hat{\theta}_i$.

This may not appear on the surface to be the same as the above-described error-modified bootstrap. However, this is a special case of the error-modified bootstrap. Here there is a single datum for the quantity in question. Since this quantity is a function of the given parameters, the distribution of the quantity is a function of the distributions of the parameters. Therefore this parameter bootstrap is really the error-modified bootstrap in the case of a single datum.

2.4 Error Analysis

Error analysis (meaning here estimation of uncertainties) was accomplished through different means, depending on the application. Here we describe our methods for error analysis for SED parameters, seed factor parameters, and the various other distributions related to the seed factor as appropriate.

2.4.1 SED Error Analysis.

Error analysis of the parameters of the SED models was accomplished via application of Wilk's Theorem through the profile-likelihood method. For a given parameter, $\hat{\theta_n}$, for which the uncertainties were to be calculated, the log-likelihood of a given SED model was calculated for a sample of values, $\{\theta_n^*\}$, with all other parameters fixed at their best-fit values. This sample of log-likelihoods was then transformed by subtracting from all the log-likelihood values the difference of the best-fit log-likelihood and the target delta-log-likelihood (as given by Wilk's Theorem). This transformation was performed so that a root-finding method could be employed to find the uncertainties. This transformed sample was then interpolated using `scipy.interpolate.splrep`, and `scipy.interpolate.sproot` was used to find the roots.

Error analysis of the peak frequencies and peak fluxes was performed differently depending on the SED model preferred for a given SED. In the case of log-parabolic models, the peak frequency and peak flux are parameters of the model. Therefore the uncertainties of the peak frequency and peak flux for any log-parabolic model was calculated using the profile-likelihood implementation described above.

In the case of third-degree polynomials, however, the peak frequency and peak flux are not parameters of the model, and are instead calculated from the model after fitting. The uncertainties therefore cannot be calculated directly using the profile-likelihood method. A bootstrapping procedure (parameter bootstrap, as described in 2.3) was therefore employed to estimate the uncertainties. This method relied on first estimating the uncertainties of the model parameters through the profile-likelihood method, and then creating a large sample (10^6 for a pure third-degree polynomial and 10^7 for a third-degree polynomial with added big-blue bump) of peak values calculated for parameter tuples drawn randomly from binormal distributions as defined by the parameter uncertainties calculated beforehand. A kernel-density estimate was then created from the bootstrapped peak values using `sklearn.neighbors.KernelDensity` to provide an estimate of the log-likelihood function of each peak value. The bandwidth used for the kernel density estimate in this method was chosen based on testing. This log-likelihood estimate was then sampled and interpolated in the same way as for the model parameter error analysis, and roots were found to estimate the uncertainties of the peak values.

2.4.2 Seed Factor Error Analysis.

The uncertainties for the seed factor calculated for each individual SED were estimated using the error-modified bootstrap. The uncertainty on the seed factor population median was estimated using a bootstrap method (parameter bootstrap, as described in 2.3) on the individual seed factors. This was done by sampling the observed seed factor distribution with replacement, drawing, from each seed factor selected in a given sample, a value from the distribution defined by its uncertainties. In effect this is a modification of the bootstrap method which incorporates the uncertainties of the measured seed factors. 1σ confidence intervals were then constructed by finding the values on either side of the peak of the median seed factor distribution at which half the probability of a normal distribution 1σ interval was enclosed (that is to say that on the upper and lower sides of the median, it was found at what point, rounding for brevity to the nearest whole number, 34% of the distribution was enclosed).

2.5 Kernel Density Estimation of the Seed Factor Distribution

A kernel density estimate was calculated for a bootstrapped sample of the observed distribution of seed factors. Bootstrap samples were calculated using the seed factor uncertainties to better approximate the distribution. The bandwidth of the kernel density estimate was chosen using Silverman's Rule [35]. Silverman's Rule gives a bandwidth which approximates the bandwidth which would minimize the mean integrated squared error. Silverman's Rule gives a bandwidth,

$$h = \left(\frac{4\sigma^5}{3n}\right)^{1/5} \tag{17}$$

where σ is the sample standard deviation, and n is the number of data. In our method, the standard deviation of the bootstrapped distribution was taken as σ (being an estimate of the population standard deviation), and the number of observed data was taken as n. We used a Gaussian kernel for the kernel density estimate.

References

[1] Tavecchio, F. & Ghisellini, G. The spectrum of the broad-line region and the high-energy emission of powerful blazars. *Monthly Notices of the Royal Astronomical Society* **386**, 945–952 (2008). URL http://adsabs.harvard.edu/abs/2008MNRAS.386..945T.

[2] Sikora, M., Stawarz, L., Moderski, R., Nalewajko, K. & Madejski, G. M. Constraining Emission Models of Luminous Blazar Sources. *The Astrophysical Journal* **704**, 38 (2009). URL https://ui.adsabs.harvard.edu/abs/2009ApJ...704...38S/abstract.

[3] Suganuma, M. *et al.* Reverberation Measurements of the Inner Radius of the Dust Torus in Nearby Seyfert 1 Galaxies. *The Astrophysical Journal* **639**, 46–63 (2006). URL http://adsabs.harvard.edu/abs/2006ApJ...639...46S.

[4] Pozo Nuñez, F. *et al.* Dust reverberation-mapping of the Seyfert 1 galaxy WPVS48. *Astronomy and Astrophysics* **561**, L8 (2014). URL http://adsabs.harvard.edu/abs/2014A%26A...561L...8P.

[5] Kishimoto, M. *et al.* Mapping the radial structure of AGN tori. *Astronomy and Astrophysics* **536**, A78 (2011). URL http://adsabs.harvard.edu/abs/2011A%26A...536A..78K.

[6] Hao, H. *et al.* Hot-dust-poor Type 1 Active Galactic Nuclei in the COSMOS Survey. *The Astrophysical Journal* **724**, L59 (2010). URL https://ui.adsabs.harvard.edu/abs/2010ApJ...724L..59H/abstract.

[7] Hao, H., Elvis, M., Civano, F. & Lawrence, A. Hot-dust-poor Quasars in Mid-infrared and Optically Selected Samples. *The Astrophysical Journal* **733**, 108 (2011). URL https://ui.adsabs.harvard.edu/abs/2011ApJ...733..108H/abstract.

[8] Maiolino, R. *et al.* Dust covering factor, silicate emission, and star formation in luminous QSOs. *Astronomy and Astrophysics* **468**, 979 (2007). URL https://ui.adsabs.harvard.edu/abs/2007A%26A...468..979M/abstract.

[9] Malmrose, M. P., Marscher, A. P., Jorstad, S. G., Nikutta, R. & Elitzur, M. Emission from Hot Dust in the Infrared Spectra of Gamma-ray Bright Blazars. *The Astrophysical Journal* **732**, 116 (2011). URL https://ui.adsabs.harvard.edu/abs/2011ApJ...732..116M/abstract.

[10] Bentz, M. C. *et al.* THE LOW-LUMINOSITY END OF THE RADIUS-LUMINOSITY RELATIONSHIP FOR ACTIVE GALACTIC NUCLEI. *The Astrophysical Journal* **767**, 149 (2013). URL http://stacks.iop.org/0004-637X/767/i=2/a=149?key=crossref.a77dd784ef4cb027eabc65407221e0d2.

[11] Ghisellini, G. & Tavecchio, F. Canonical high-power blazars. *Monthly Notices of the Royal Astronomical Society* **397**, 985–1002 (2009). URL http://adsabs.harvard.edu/abs/2009MNRAS.397..985G.

[12] Nenkova, M., Sirocky, M. M., Nikutta, R., Ivezić, Ž. & Elitzur, M. AGN Dusty Tori. II. Observational Implications of Clumpiness. *The Astrophysical Journal* **685**, 160–180 (2008). URL http://stacks.iop.org/0004-637X/685/i=1/a=160.

[13] Abdo, A. A. *et al.* THE SPECTRAL ENERGY DISTRIBUTION OF *FERMI* BRIGHT BLAZARS. *The Astrophysical Journal* **716**, 30–70 (2010). URL http://stacks.iop.org/0004-637X/716/i=1/a=30?key=crossref.935a55ae61932f774af24599abe5dcf9.

[14] Abdo, A. *et al.* VizieR Online Data Catalog: SED of Fermi bright blazars (2010). URL https://doi.org//10.26093/cds/vizier.17160030.

[15] Demleitner, M. *et al.* ADS's Dexter Data Extraction Applet. vol. 238, 321 (2001). URL http://adsabs.harvard.edu/abs/2001ASPC..238..321D.

[16] Giommi, P. *et al.* Simultaneous Planck, Swift, and Fermi observations of X-ray and -ray selected blazars. *Astronomy and Astrophysics* **541**, A160 (2012). URL http://adsabs.harvard.edu/abs/2012A%26A...541A.160G.

[17] Planck Collaboration *et al.* Planck early results. XV. Spectral energy distributions and radio continuum spectra of northern extragalactic radio sources. *Astronomy and Astrophysics* **536**, A15 (2011). URL http://adsabs.harvard.edu/abs/2011A%26A...536A..15P.

[18] Giommi, P. *et al.* VizieR Online Data Catalog: Planck + X/gamma observations of blazars (2012). URL https://doi.org//10.26093/cds/vizier.35410160.

[19] Krauß, F. *et al.* The TANAMI Multiwavelength Program: Dynamic spectral energy distributions of southern blazars. *Astronomy & Astrophysics* **591**, A130 (2016). URL http://www.aanda.org/10.1051/0004-6361/201628595.

[20] Acero, F. *et al.* *FERMI* LARGE AREA TELESCOPE THIRD SOURCE CATALOG. *The Astrophysical Journal Supplement Series* **218**, 23 (2015). URL http://stacks.iop.org/0067-0049/218/i=2/a=23?key=crossref.6a12a25cea2b8468dc5a3e407d17f75a.

[21] Ojha, R. *et al.* TANAMI: tracking active galactic nuclei with austral milliarcsecond interferometry . I. First-epoch 8.4 GHz images. *Astronomy and Astrophysics* **519**, A45 (2010). URL https://ui.adsabs.harvard.edu/abs/2010A%26A...519A..45O/abstract.

[22] Krauß, F. *et al.* VizieR Online Data Catalog: Dynamic SEDs of southern blazars - DSSB (2016). URL https://doi.org//10.26093/cds/vizier.35910130.

[23] Wehrle, A. E. *et al.* Multiwavelength Observations of a Dramatic High-Energy Flare in the Blazar 3c 279. *The Astrophysical Journal* **497**, 178–187 (1998). URL http://adsabs.harvard.edu/abs/1998ApJ...497..178W.

[24] Abdo, A. A. *et al.* Early Fermi Gamma-ray Space Telescope Observations of the Quasar 3c 454.3. *The Astrophysical Journal* **699**, 817 (2009). URL http://adsabs.harvard.edu/abs/2009ApJ...699..817A.

[25] Vercellone, S. *et al.* Multiwavelength Observations of 3c 454.3. III. Eighteen Months of Agile Monitoring of the "Crazy Diamond". *The Astrophysical Journal* **712**, 405–420 (2010). URL http://adsabs.harvard.edu/abs/2010ApJ...712..405V.

[26] Vercellone, S. *et al.* The Brightest Gamma-Ray Flaring Blazar in the Sky: AGILE and Multi-wavelength Observations of 3c 454.3 During 2010 November. *The Astrophysical Journal Letters* **736**, L38 (2011). URL http://adsabs.harvard.edu/abs/2011ApJ...736L..38V.

[27] Hayashida, M. *et al.* Rapid Variability of Blazar 3c 279 during Flaring States in 2013-2014 with Joint Fermi-LAT, NuSTAR, Swift, and Ground-Based Multiwavelength Observations. *The Astrophysical Journal* **807**, 79 (2015). URL http://adsabs.harvard.edu/abs/2015ApJ...807...79H.

[28] Hayashida, M. *et al.* The Structure and Emission Model of the Relativistic Jet in the Quasar 3c 279 Inferred from Radio to High-energy -Ray Observations in 2008-2010. *The Astrophysical Journal* **754**, 114 (2012). URL http://adsabs.harvard.edu/abs/2012ApJ...754..114H.

[29] Pacciani, L. *et al.* The 2009 December Gamma-ray Flare of 3c 454.3: The Multifrequency Campaign. *The Astrophysical Journal Letters* **716**, L170–L175 (2010). URL http://adsabs.harvard.edu/abs/2010ApJ...716L.170P.

[30] Abdo, A. A. *et al.* Gamma-ray Light Curves and Variability of Bright Fermi-detected Blazars. *The Astrophysical Journal* **722**, 520–542 (2010). URL http://adsabs.harvard.edu/abs/2010ApJ...722..520A.

[31] Ghisellini, G., Tavecchio, F., Foschini, L. & Ghirlanda, G. The transition between BL Lac objects and flat spectrum radio quasars: FSRQs and BL Lacs. *Monthly Notices of the Royal Astronomical Society* **414**, 2674–2689 (2011). URL https://academic.oup.com/mnras/article-lookup/doi/10.1111/j.1365-2966.2011.18578.x.

[32] Corana, A., Marchesi, M., Martini, C. & Ridella, S. Minimizing Multimodal Functions of Continuous Variables with the "Simulated Annealing" Algorithm. *ACM Trans. Math. Softw.* **13**, 262–280 (1987). URL http://doi.acm.org/10.1145/29380.29864.

[33] Goffe, W. L., Ferrier, G. D. & Rogers, J. Global optimization of statistical functions with simulated annealing. *Journal of Econometrics* **60**, 65–99 (1994). URL http://www.sciencedirect.com/science/article/pii/0304407694900388.

[34] Domínguez, A. *et al.* Extragalactic background light inferred from AEGIS galaxy-SED-type fractions. *Monthly Notices of the Royal Astronomical Society* **410**, 2556–2578 (2011). URL http://adsabs.harvard.edu/abs/2011MNRAS.410.2556D.

[35] Silverman, B. W. *Density estimation for statistics and data analysis.* No. 26 in Monographs on statistics and applied probability (Chapman & Hall/CRC, Boca Raton, 1998).
